# Innovative Bacterial Colony Detection: Leveraging Multi-Feature Selection with the Improved Salp Swarm Algorithm

**DOI:** 10.3390/jimaging9120263

**Published:** 2023-11-28

**Authors:** Ahmad Ihsan, Khairul Muttaqin, Rahmatul Fajri, Mursyidah Mursyidah, Islam Md Rizwanul Fattah

**Affiliations:** 1 Department of Informatics, Faculty of Engineering, Universitas Samudra, Langsa 24416, Aceh, Indonesia; khairulmuttaqin@unsam.ac.id; 2 Department of Chemistry, Faculty of Engineering, Universitas Samudra, Langsa 24416, Aceh, Indonesia; rahmatulfajri@unsam.ac.id; 3 Department of Multimedia Engineering Technology, Politeknik Negeri Lhokseumawe, Kota Lhokseumawe 24301, Aceh, Indonesia; mursyidah@pnl.ac.id; 4 Centre for Technology in Water and Wastewater, School of Civil and Environmental Engineering, Faculty of Engineering and IT, University of Technology Sydney, Ultimo, NSW 2007, Australia; islammdrizwanul.fattah@uts.edu.au

**Keywords:** bacterial colony, multi-feature selection, classification accuracy, improved salp swarm algorithm

## Abstract

In this paper, we introduce a new and advanced multi-feature selection method for bacterial classification that uses the salp swarm algorithm (SSA). We improve the SSA’s performance by using opposition-based learning (OBL) and a local search algorithm (LSA). The proposed method has three main stages, which automate the categorization of bacteria based on their unique characteristics. The method uses a multi-feature selection approach augmented by an enhanced version of the SSA. The enhancements include using OBL to increase population diversity during the search process and LSA to address local optimization problems. The improved salp swarm algorithm (ISSA) is designed to optimize multi-feature selection by increasing the number of selected features and improving classification accuracy. We compare the ISSA’s performance to that of several other algorithms on ten different test datasets. The results show that the ISSA outperforms the other algorithms in terms of classification accuracy on three datasets with 19 features, achieving an accuracy of 73.75%. Additionally, the ISSA excels at determining the optimal number of features and producing a better fit value, with a classification error rate of 0.249. Therefore, the ISSA method is expected to make a significant contribution to solving feature selection problems in bacterial analysis.

## 1. Introduction

Microorganisms, also known as microbes, can pose a significant threat to human health and ecological systems. One of the most significant risks is their role in causing a variety of human diseases, including dysentery, tuberculosis, pneumonia, sepsis, typhus, diarrhea, and tetanus [1]. Furthermore, the negative effects of microorganisms also extend to agriculture, particularly the process of soil desalination [2]. One type of microbe that poses a threat is bacteria in water [3]. The World Health Organization (WHO) estimates that 2 billion people worldwide drink feces-contaminated water, which causes an estimated 525,000 deaths from diarrhea each year in children under five. According to the World Bank, poor water quality can limit economic growth, constrain human development, and disrupt food production [4]. 

Bacteria are microscopic organisms that are invisible to the naked eye. They are typically identified using a microscope, but this process can be time-consuming and laborious. Automatic identification of bacteria in water is a promising new technique that could improve the speed and accuracy of bacterial identification. Traditional laboratory-based methods for identifying bacteria rely heavily on the expertise of laboratory technicians, which can lead to significant variations in results. Additionally, this laboratory-centric approach requires resource-intensive protocols that are expensive and time-consuming [5]. Therefore, an automated bacterial identification system is required that can leverage artificial intelligence (AI) to accurately and efficiently identify bacteria. 

AI systems have become an attractive choice for bacterial identification [4]. One of the critical steps in AI-based bacterial identification is feature extraction, which involves identifying and extracting distinctive features of bacteria that can be used for classification. There are three general categories in feature extraction for the automatic classification of bacteria: first, the category of statistical approaches; second, machine-learning techniques such as artificial neural networks and support vector machines (SVMs); and third, geometric feature extraction from input images [6]. In practice, a combination of feature extraction techniques may be used to achieve the best results for bacterial classification.

Panicker et al. [7] proposed an approach for the automatic detection of tuberculosis bacteria from microscopic images of speckled sputum using geometric features, including color averaging of the extracted images. This study shows that color features have discriminatory properties and have a significant effect on improving image recognition. On the other hand, Luo et al. [8] used segmented images to extract the shape and size of microorganisms and then classify them based on their type. Ernest Bonah [9] introduced an approach to identifying food-related pathogens based on the dispersal patterns of bacterial colonies. They use a group of features that are analyzed using data mining algorithms to reduce feature dimensions, then adopt an SVM in the classification stage. 

Convolutional neural networks (CNNs) have also become an interesting method for feature extraction and classification of bacterial images [10,11,12,13,14]. In addition to pre-processing approaches such as segmentation, CNNs also utilize data augmentation and transfer learning to improve the ability to classify and identify images. Despite having a high success rate in classification, this approach requires significant computational power and is often time-consuming. In addition, the success of CNNs in the classification task requires computer hardware with a high configuration, such as a graphics processing unit (GPU) or multi-CPU, to complete the task properly. 

Zieliński et al. [15] employed the deep convolutional neural networks (CNNs) technique for the recognition of bacterial genera and species. Within this investigation, a CNN was utilized to extract distinctive attributes, while the classification phase involved support vector machines (SVMs). This approach is associated with limitations, including substantial resource consumption, a need for enhanced accuracy, and struggles in discerning-colored bacterial images. These complexities underscore the impetus for continued research in advancing more effective strategies for both feature extraction and classification.

In recent years, various types of orthogonal moments have been developed to describe and extract features from color images, including quaternion moments [16,17,18,19,20] and multi-channel moments [21]. Orthogonal functions with an order of fractions have been shown to have a better ability to represent the fine details of a given function compared to functions that have an order of integers. The interesting characteristics of fractional successive orthogonal moments have led the authors to develop Hu moment invariants and Zernike moments, which are used to extract fine features from colored images of bacteria. 

This study applies feature selection techniques to reduce the number of extracted features and remove irrelevant ones [22,23,24,25]. One popular approach is the swarm intelligence algorithm [26]. The salp swarm algorithm (SSA) is a swarm intelligence algorithm that has been shown to be effective in identifying the most informative subset of features [27]. Several researchers have improved the SSA for feature selection. For example, Miodrag Zivkovic [28] developed an improved SSA that achieved better results on 21 benchmark datasets. Chaabane et al. [29] used SSA to optimize the selection of feature weights before training a machine-learning model for blind modulation identification. This study uses salp swarm optimization to minimize the misclassification rate in blind modulation identification. Shikai Wang et al. [30] developed a color image segmentation method using SSA to find the optimal threshold set in the multilevel thresholding process. This study adopts the salp movement behavior to find the optimal threshold set in the multilevel thresholding process. Xiaojun Xie et al. [31] developed an optimal feature selection combination strategy based on SSA for plant disease detection. While the SSA has the potential to select image features, it has some drawbacks, such as the difficulty of setting parameters and the risk of stagnation. In addition to SSA, there is another technique that can be used for feature selection, namely particle swarm optimization (PSO). The PSO algorithm has a simple number of features and fast convergence speed. However, PSO has several limitations, such as population diversity and local optima [32]. Furthermore, PSO does not effectively work for large-scale problems [33]. To address these issues, this study uses a feature selection technique based on the improved salp swarm algorithm (ISSA) [34]. The ISSA incorporates two significant enhancements over the SSA: it employs opposition-based learning (OBL) during SSA initialization to augment population diversity within the search space and incorporates a local search algorithm to enhance exploitation. The ISSA was chosen as a means to optimize the feature selection process to improve the quality of relevant features and classification accuracy. 

This paper proposes an advanced multi-feature selection method for bacterial colonies, leveraging the integration of the improved salp swarm algorithm (ISSA), opposition-based learning (OBL), and a local search algorithm (LSA). The primary goal is to improve the accuracy and efficiency of bacterial identification using advanced AI techniques. The proposed method will be compared to other existing algorithms, such as the salp swarm algorithm (SSA) and particle swarm optimization (PSO), on 19 different bacterial features to assess its performance. Additionally, this study will highlight the ISSA algorithm’s significance in improving feature selection and classification accuracy, addressing the current limitations of bacterial identification techniques. The scope also includes a comparative analysis of the ISSA algorithm’s performance against other prevalent methods, highlighting its advantages and potential contributions to the field of microbial identification and classification.

## 2. Materials and Methods

This section provides a comprehensive overview of all the experimental procedures conducted in this study. Specifically, it details the microbiological methods used to acquire the image database, the pre-processing, segmentation, feature extraction, feature selection, and image classification techniques employed, and presents step-by-step flowcharts to illustrate each step.

### 2.1. Pre-Processing

Image pre-processing is a stage of image quality enhancement that precedes further analysis. The first step in pre-processing is to convert the image from RGB color space to grayscale. This step reduces the computational complexity of subsequent processing by using a single intensity channel. Additionally, the grayscale representation can facilitate a clearer interpretation of image features, which is essential for further analysis. This is performed using the National Television System Committee (NTSC) formula, namely 0.299* red component + 0.587* green component + 0.114* blue component. The second step applies median filtering to reduce noise in the image without losing contrast. To overcome the possibility of uneven lighting, a top-hat transformation with a radius of 200 pixels is carried out and uses a single disc-shaped structural element. The third step, contrast adjustment, is the process of changing the intensity range of pixels in an image to make the image sharper, clearer, and more detailed so that information in the image can be more easily recognized and understood. In this step, the image is normalized using Equation (1); *p_adjusted_* is the replaced pixel value, *p* is the current pixel value, *p_min_* is the minimum pixel value, *NC* is the normalization coefficient, and *MI* is the maximum intensity value of the image.

(1) $$\sum_{p_{n}}\begin{array}{c} p_{adjusted=\frac{p-p_{min}}{NC}\;x\;MI\;} \end{array}$$

**Figure 1:**
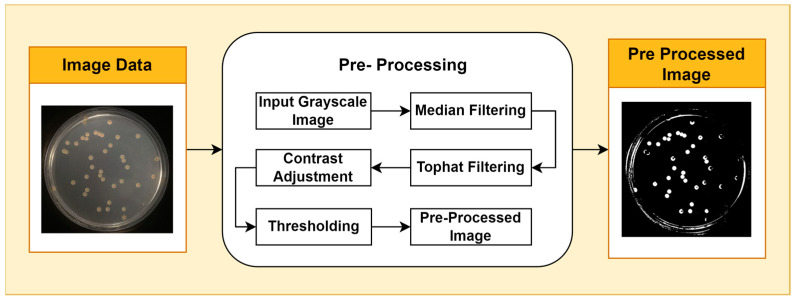
Pre-Processing Stages.

To segment the object from the background, a binary thresholding technique is applied. In this process, each pixel in the image is converted to a binary value (black or white) based on a predefined threshold value. Binary thresholding is used to segment the object from the background by converting each pixel in the image to a binary value (black or white) based on a predefined threshold. Pixels with an intensity above the threshold are considered white, while pixels with an intensity below the threshold are considered black. This stage isolates the foreground from the background, allowing for the isolation of objects of interest for further analysis. By converting each pixel to a binary value, this process effectively divides the image into distinct parts and extracts relevant information related to specific features of interest. The pre-processing stages are shown in Figure 1. 

### 2.2. Segmentation 

**Figure 2:**
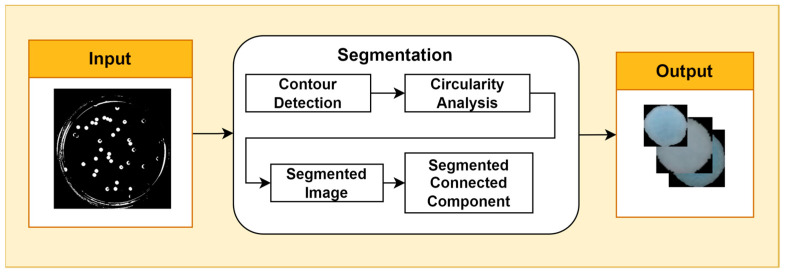
Segmentation Stage.

Image segmentation is the process of partitioning an image into multiple regions with semantically meaningful or visually distinct characteristics. It is a fundamental task in computer vision with a wide range of applications, such as object detection, tracking, and classification. One common approach to image segmentation is contour detection, which aims to identify and extract the boundaries that separate objects in the image. A popular contour detection algorithm is Canny’s edge detector, which uses a multi-stage process to identify edges with high probability and low localization error. Once the contours of bacterial cells have been detected, the Douglas–Peucker algorithm can be used to simplify them by approximating them with a sequence of line segments. The epsilon parameter controls the maximum distance allowed between a point on the original contour and the corresponding point on the approximated contour. A smaller epsilon value will result in a more detailed approximation, while a larger epsilon value will result in a more simplified approximation. The choice of epsilon value depends on the desired level of contour detail. Circularity analysis is a technique used to measure the extent to which an object or structure in an image approaches a circular shape. It is often used in biological image processing to identify and segment cells. A variety of circularity metrics can be used, such as the aspect ratio, circularity index, and Feret’s diameter. The choice of metric depends on the specific application. In this study, circularity analysis was performed using the circular Hough transform (Figure 2). 

### 2.3. Feature Extraction

Feature extraction is an essential step in bacterial image analysis, as it allows us to identify, classify, and characterize different types of bacteria based on their visual features. There are many different types of features that can be extracted from bacterial images, including geometric features, color features, texture features, and moment features.

Geometric features provide information about the shape and size of bacterial cells, such as area, perimeter, eccentricity, circularity, roughness, convex area, and center of mass (centroid x and centroid y). Color features are measured using color metrics such as hue, saturation, and value (HSV). Texture features describe the visual patterns in an image, such as contrast, energy, homogeneity, dissimilarity, correlation, and opacity. Moment features are mathematical representations of the geometric properties of objects in an image.

**Figure 3:**
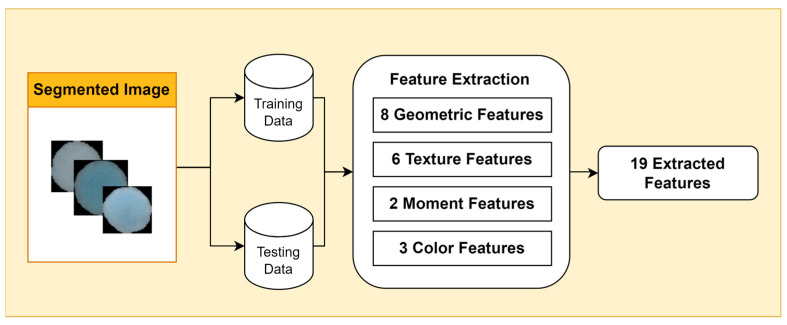
Feature Extraction Stages.

In this study, we focus on two types of moment features: Hu moments and Zernike moments (Figure 3). Hu moments are invariant to translation, rotation, and scale, making them useful for feature extraction from bacterial images, which can vary in size and orientation. Zernike moments are more sensitive to small changes in image structure, making them useful for discriminating between different types of bacteria.

### 2.4. Feature Selection

#### 2.4.1. Salp Swarm Algorithm 

The salp swarm algorithm is a recently developed nature-inspired metaheuristic algorithm that mimics the particular swarming behavior of marine salps. Salps usually live in groups and often form shoals called salp chains. The first salp is referred to as the leader, while the others are followers. In the mathematical model of the salp, the position of the salp leader is updated using Equation (2).

(2) $$x_{j}^{1}=\left\{\begin{array}{c} F_{j}+c_{1}\left(\left(ub_{j}-lb_{j}\right)c_{2}+lb_{j}\right)\;c_{3}\geq 0.5\\ F_{j}-c_{1}\left(\left(ub_{j}-lb_{j}\right)c_{2}+lb_{j}\right)\;c_{3}<0.5 \end{array}\right.$$

where the value $\;x_{j}^{1}$ indicates the position of the leader in the salp chain, and ${\;F}_{j}$ shows the position of the food source in the *j* dimension. Moreover, it represents the lower bound value of the *j* dimension. In $\;b_{j}$ represents the upper bound value of *j*. Then, *c*_1_, *c*_2_, and *c*_3_ represent random values; *c*_1_ is the most important controlling parameter in SSA because it is responsible for maintaining the balance between exploration and exploitation in SSA. Equation (3) is used to calculate the value of parameter *c*.

(3) $$c_{1}=2{e^{-\left(\frac{4l}{L}\right)}}^{2}$$

In Equation (3),  l represents the current iteration, and  L  represents the maximum number of iterations of the algorithm. Parameter values of $c_{2}$ and $c_{3}$ denote random values in the range [0, 1]. The positions of the followers in the salp chain are updated using Equation (4),

(4) $$x_{j}^{1}=\frac{1}{2}\left(x_{j}^{i\;}+x_{j}^{i-1}\right)$$

where  i ≥2 and $x_{j}^{i\;}$ is the position of  i follower in dimension  j search space. The SSA algorithm is shown in Algorithm 1.
**Algorithm 1** Pseudocode of the SSA algorithm
*initialize the salps’ positions xi (i = 1, 2, …, n)*
*while (t < max iterations)*

*determine the fitness value of each salp*

*F = best salp ((search-agent)*

*Update the value of c parameter using Equation (2)*

*for every salp (xi)*


*if (i == 1)*



*Update leader position using Equation (1)*


*Else*



*Update follower position using Equation (3)*


*end if*

*end for*

*reposition the salps that go out of the search space based on the lower and upper bounds of problem variables*

*T = t + 1*
*end while*
*return F*

#### 2.4.2. Opposition-Based Learning 

OBL is an optimization technique used to improve the quality of the initial solution in a population by introducing variations to the solution. OBL operates by searching in two directions in the search space. Both directions involve the initial solution, as well as the opposite direction, which is represented by a solution that has the opposite properties. In the end, OBL chooses the best solutions from all the existing ones. 

Opposite numbers, denoted as x, are defined as real numbers on the interval. The opposite number is x, denoted as follows and determined using Equation (5),

(5) $$\tilde{x}=lb+ub-x$$

Equation (5) can be generalized to apply in a multidimensional search space. Therefore, Equations (6) and (7) will represent the position of each search agent and its opposite position in order to generalize this as follows:

(6) $$x=\left[x_{1},x_{2},x_{3},\;\ldots \ldots ..,x_{D}\;\right]$$

(7) $$\tilde{x}=\left[{\tilde{x}}_{1},{\tilde{x}}_{2},{\tilde{x}}_{3},\;\ldots \ldots ..,{\tilde{x}}_{D}\;\right]$$

The values of all the inner elements$\;\tilde{x}$ will be determined using Equation (8):

(8) $$\tilde{x_{j}}=lb_{j}+{ub}_{j}-x_{j}\;j\;=\;1,\;2,\;3,\;\ldots ,\;D$$

In the optimization based on opposed populations approach, the fitness function is represented as *f*(). Therefore, if the fitness value $f(\tilde{x})\;$ of the opposite solution is superior to $f\left(x\right)\;$, that of the initial solution *x*, then x = $\tilde{x}$; if not,  x = x.

The integration of OBL (opposition-based learning) with SSA (salp swarm algorithm) involves the following steps: initializing the salp positions *x* as $x_{i}\;$ and determining the opposite positions of the salp population OX as $\tilde{x_{i}}$, where (*i* = 1, 2, …, *n*); subsequently, the n strongest salps selected from {X ∪ OX} will constitute the new initial population for SSA. 

#### 2.4.3. Local Search Algorithm 

The developed local search algorithm (LSA) is shown in Algorithm 2. LSA will be called at the end of each iteration in SSA to improve the best solution. Initially, LSA stores the best solution value obtained from SSA in the last SSA iteration into the Temp variable. LSA performs a number of iterations to increase temperature. In each LSA iteration, the LSA randomly selects three features from the Temp. LSA rearranges the selected features based on their values. In addition, LSA will determine the fitness value of the new solution; if it is better than the initial fitness value, it will be set to Temp; otherwise, it will remain unchanged.
**Algorithm 2** Pseudocode of the LSA algorithm
*Temp = F (where F represents the current best solution at end of SSA’s current iteration)*
*Lt = 1 (Lt is a variable used to store the current iteration of local search algorithm)*
*while (Lt < maximum number of local iterations)*

*Randomly select three features from Temp*


*if selected-feature == 1 (1 means the feature is selected and 0 means not selected)*



*selected-feature = 0*


*Else*



*selected-feature = 1*


*end if*

*Calculate the fitness value of Temp*


*if f(Temp) < f(F)*



*F = Temp*


*end if*


*Lt = Lt + 1*
*end while*
*return F*

#### 2.4.4. Improved Salp Swarm Algorithm 

**Figure 4:**
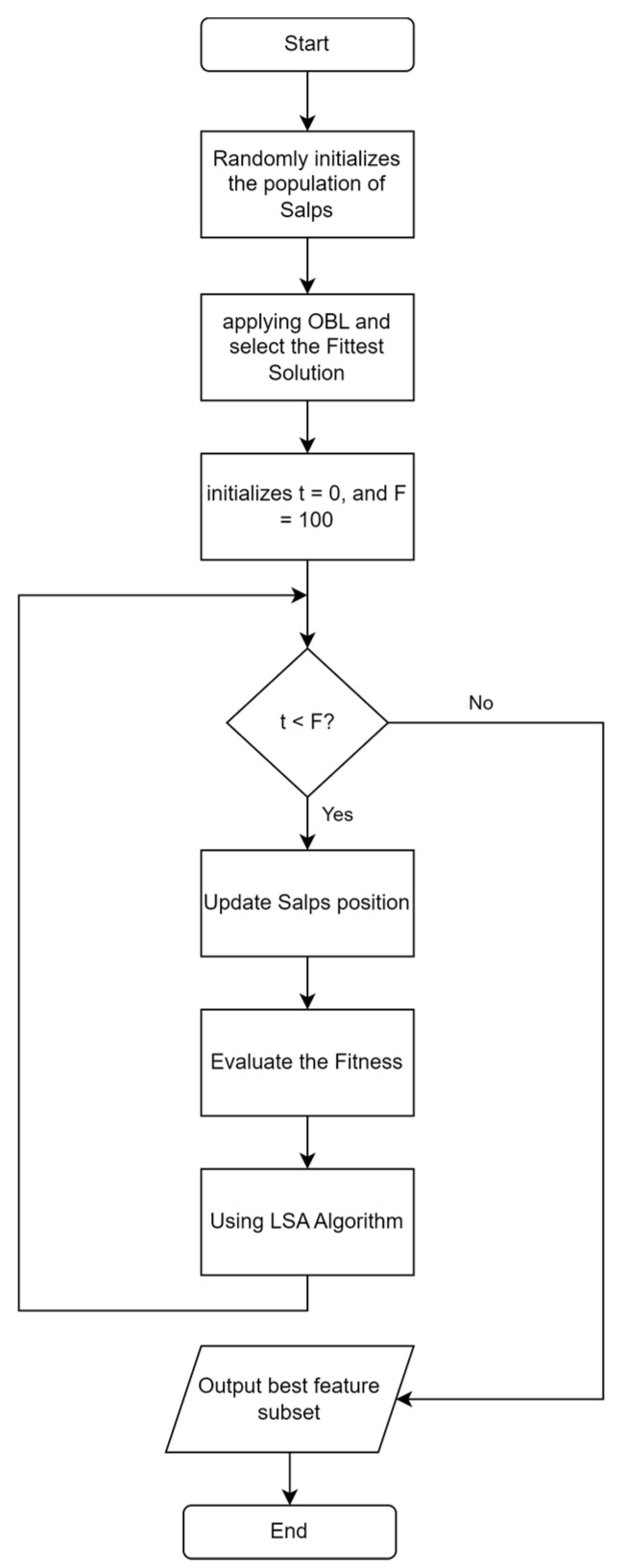
Flowchart ISSA algorithm based on OBL and LSA algorithms.

**Figure 5:**
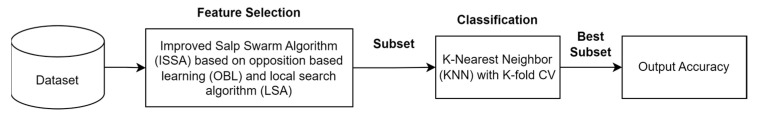
The Second Stage of Feature Selection.

ISSA is an improvement on the SSA algorithm. The stages of the proposed ISSA algorithm are shown in Figure 4. The steps of the ISSA algorithm include initializing SSA by generating the number of salps based on population size and selecting a feature subset randomly from the entire feature set. This is followed by the application of OBL to find the opposite solution from each initial solution and the reverse solution, which is calculated based on the KNN classification accuracy error, as shown in Figure 5. Additionally, SSA gives a t value to the best solution that OBL selects as having the lowest classification accuracy error. Depending on whether the salp is the leader or a follower in the salp chain, Equation (2) or (4) updates the position of each salp after that. Fitness evaluation is performed by determining the fitness values of all salps, and these values are updated if a better solution is found. The LSA algorithm is applied to the t best solution to find a better solution, and the t value is updated if a better solution is found. The ISSA algorithm is repeated in iterations. ISSA returns the best solution that represents the best feature subset. In the testing phase, the features selected for the best solution are used to evaluate ISSA’s performance on the test dataset. 

The ISSA algorithm is compared to the standard SSA algorithm and the particle swarm optimization (PSO) algorithm. The population size utilized by all algorithms is 10, the maximum number of iterations for each optimization algorithm is 100, and the number of LSA iterations is fixed at 10. An indicator to evaluate the performance of the optimization algorithm is the classification accuracy using KNN classification.

## 3. Results and Discussion 

Given the growing concern about the adverse impact of microorganisms on human health and ecological systems, this study aims to propose an advanced multi-feature selection method for bacterial colonies. This method incorporates the salp swarm algorithm (SSA), opposition-based learning (OBL), and local search algorithm (LSA), with the primary objective of enhancing the accuracy and efficiency of bacterial identification through sophisticated artificial intelligence techniques. This study will conduct a comparison between the proposed method and other existing algorithms, such as the salp swarm algorithm (SSA) and particle swarm optimization (PSO), across 19 distinct bacterial features to evaluate its performance. Furthermore, this research will underscore the significance of the ISSA algorithm in refining feature selection and classification accuracy while addressing current constraints within bacterial identification techniques. The research scope also encompasses an analysis that compares the performance of the ISSA algorithm with other prevalent methods, emphasizing its benefits and potential contributions in the realm of microbial identification and classification.

### 3.1. Dataset 

To evaluate and validate the performance of the ISSA algorithm compared to other algorithms, the bacterial benchmark dataset from the CINATE repository (Center for Innovation and Technological Support, ESB, UCP) was used [35]. Details of the datasets used can be seen in Table 1.

### 3.2. Parameter Setting 

All experiments were conducted using a 10-fold cross-validation method. This validation method divides the data into 10 parts, with 70% of the data used for training and 30% used for testing. The experiment was repeated 10 times, and the results of the 10 trials were averaged to produce the performance indicators. Therefore, the results listed in Table 2 reflect the average value of 100 iterations in terms of accuracy, suitability, and selected features.

### 3.3. Results and Analysis

This section presents a comprehensive overview of the key findings and results of the conducted experiments. Specifically, this study compares the performance of SSA, PBO, and ISSA on bacterial colony images. The evaluation of ISSA’s efficacy against other algorithms is based on three key metrics: classification accuracy, the number of selected features, and the match value. Table 2 summarizes the results of the comparative analysis of SSA, PSO, and ISSA. ISSA outperforms SSA and PSO in classification accuracy on three datasets with 19 features, achieving an accuracy of 73.75%. Additionally, ISSA selects a smaller number of features than SSA and PSO in all 10 trials. In addition to accuracy, ISSA also demonstrates superior performance in match value, with a significantly lower classification error rate of 0.2623 compared to SSA. These results highlight ISSA’s significant improvement over the original SSA algorithm in terms of classification accuracy, feature selection, and match value.

**Figure 6:**
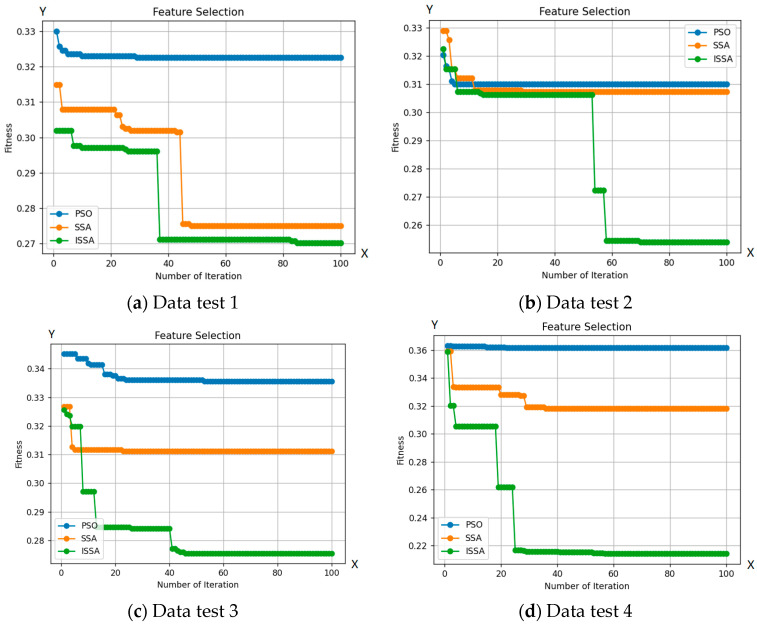

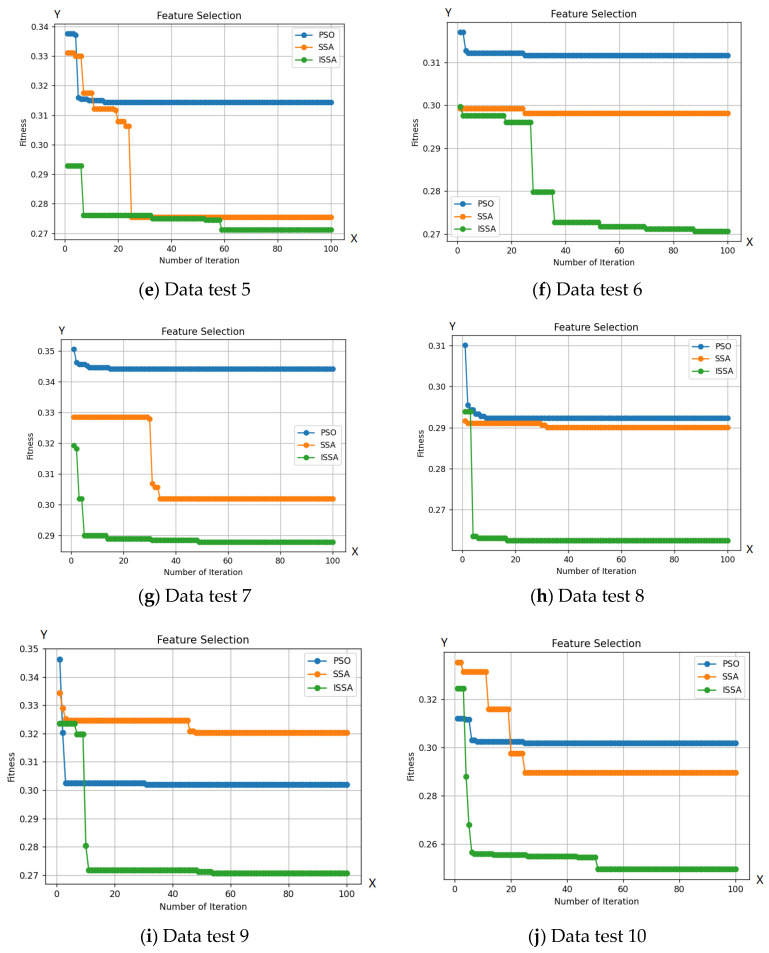
Comparison between the ISSA, PSO, and SSA methods with 10 test data.

Figure 6 demonstrates ISSA’s superior exploration capability compared to other optimization algorithms. This is evident in ISSA’s ability to access regions of the search space that are inaccessible to other algorithms, indicating its superior ability to maintain a diverse set of solutions. Additionally, Table 2 shows that ISSA consistently achieves better fit values than other algorithms, demonstrating its ability to avoid local optima. Finally, ISSA can select a smaller number of features than other optimization algorithms, further demonstrating its exploration capability. 

Based on the number of features selected by ISSA, as seen in Table 2, ISSA demonstrates superior exploration capabilities compared to the SSA and PSO algorithms. This is further strengthened by the selection of a smaller number of features from the 10 included test data. Out of the 19 features tested on 10 test data, ISSA is able to choose 5 features to identify bacteria. The use of a more limited set of features can enhance bacterial classification. The exploration superiority of ISSA is also evident through its ability to select a smaller number of features, surpassing other optimization algorithms, as reflected in the accuracy results obtained from the 10 test data. Additionally, ISSA shows a significantly lower classification error rate in 100 iterations compared to the SSA and PSO algorithms.

### 3.4. Comparison with Other Methods

In this section, the performance of the ISSA feature selection is proposed by considering the fitness value and classification error rate compared to other published methods. For instance, research conducted by Nie et al. [36] suggests the automation of the identification and classification of bacterial colony areas using a convolutional deep belief network (CDBN). The aim of this technique is to create a profound representation of small parts of images.

Through the training process, the front and back parts of the images are distinguished with a high level of accuracy using a support vector machine (SVM). Subsequently, a convolutional neural network (CNN) is employed to detect the presence of bacteria in those images. The accuracy of ISSA KNN with the K-fold cross-validation method achieves better results than those reported by Nie and his co-authors (Table 3).

## 4. Conclusions

This study presents a multi-feature selection technique, the improved salp swarm algorithm (ISSA), for accurate bacterial colony classification. We have significantly improved the ISSA approach’s effectiveness and performance by integrating the salp swarm algorithm (SSA) with the opposition-based learning (OBL) strategy and a local search algorithm (LSA). Our research methodology is a streamlined three-stage process that enables the automated categorization of bacteria based on their distinctive features.

The ISSA technique has demonstrated outstanding performance through rigorous evaluation of a comprehensive bacterial colony feature dataset and comparative analysis with several other algorithms across ten distinct test datasets. Specifically, ISSA achieved a superior classification accuracy of 73.75% on three datasets with 19 features. Additionally, ISSA excelled at determining the optimal feature count, resulting in a significantly reduced classification error rate of 0.249. These numerical results demonstrate the ISSA technique’s robustness and effectiveness in addressing the complex challenges of bacterial feature selection and classification.

While the ISSA technique has the potential to revolutionize bacterial colony classification, certain limitations must be considered. The ISSA algorithm’s computational complexity and potential constraints in scaling the methodology for larger datasets highlight the need for further optimization and resource allocation. Additionally, practical implementation of ISSA in real-time applications may require additional fine-tuning to maximize its efficiency and applicability.

Overall, the ISSA technique represents a significant advancement in bacterial colony classification, offering improved accuracy and efficiency while addressing the computational complexities of the classification task. By acknowledging the limitations and leveraging the strengths of the proposed technique, this study contributes to the ongoing efforts to refine bacterial analysis methodologies for enhanced scientific and practical applications.

## Figures and Tables

**Table 1:** Details of the datasets used.

No.	Dataset	Number of Features	Number of Samples
1	*E. coli* bacteria	19	427
2	*S. aureus* bacteria	19	371
3	*P. aeruginosa* bacteria	19	458

**Table 2:** Comparison between ISSA, PSO, and SSA methods based on average accuracy, average number of selected features, and average fit in 10 trials.

Data	Accuracy			Number of Selected Feature			Fitness		
	PSO	SSA	ISSA	PSO	SSA	ISSA	PSO	SSA	ISSA
Data Test 1	67.686	72.489	**72.925**	5	5	**4**	0.323	0.274	**0.270**
Data Test 2	68.995	69.432	**74.672**	6	9	**6**	0.310	0.307	**0.254**
Data Test 3	66.375	68.995	**72.489**	5	8	**6**	0.335	0.311	**0.276**
Data Test 4	63.755	68.122	**78.602**	5	5	**5**	0.361	0.318	**0.214**
Data Test 5	68.558	72.489	**72.925**	6	6	**6**	0.314	0.275	**0.271**
Data Test 6	68.995	70.305	**72.925**	9	8	**5**	0.311	0.298	**0.270**
Data Test 7	65.502	69.868	**71.179**	5	7	**5**	0.344	0.301	**0.287**
Data Test 8	70.742	71.179	**73.799**	5	9	**6**	0.292	0.290	**0.262**
Data Test 9	69.868	68.122	**72.925**	7	9	**5**	0.301	0.320	**0.270**
Data Test 10	69.868	71.179	**75.109**	7	8	**6**	0.301	0.289	**0.249**
**Average**	**68.034**	**70.218**	**73.755**	**6**	**7**	**5**	**0.319**	**0.298**	**0.262**

**Table 3:** The comparison with other bacteria species recognition methods.

	CNN	SIFT + KNN	SIFT + SVM	ISSA + KNN
**ACC (%)**	0.6210	0.4967	0.5400	0.7375

## Data Availability

The data presented in this study are openly available in FigShare at https://doi.org/10.6084/m9.figshare.20109377.v2 (accessed on 15 August 2023), reference number [35].
